# Analysis of Undesirable Sequelae of Sentinel Node Surgery in Breast Cancer Patients – a Prospective Cohort Study

**DOI:** 10.1007/s12253-017-0306-3

**Published:** 2017-09-16

**Authors:** Dominika Kozak, Iwona Głowacka-Mrotek, Tomasz Nowikiewicz, Zygmunt Siedlecki, Wojciech Hagner, Magdalena Sowa, Wojciech Zegarski

**Affiliations:** 1 Department of Surgical Oncology, Oncology Center in Bydgoszcz, Collegium Medicum of the Nicolaus Copernicus University in Torun, Bydgoszcz, Poland; 2 Department of Rehabilitation, Collegium Medicum of the Nicolaus Copernicus University in Torun, Maria Curie-Skłodowskiej Street 9, 85-094 Bydgoszcz, Poland; 3 Department of Neurosurgery, Collegium Medicum of the Nicolaus Copernicus University in Torun, Bydgoszcz, Poland; 4 Department of Laser Therapy and Physiotherapy, Collegium Medicum of the Nicolaus Copernicus University in Bydgoszcz, Bydgoszcz, Poland

**Keywords:** Breast cancer, Sentinel lymph node, Undesirable sequelae

## Abstract

Use of sentinel lymph node biopsy limits the frequency and severity of sequelae of surgical treatment. However, the procedure itself may not be completely free of complications. The goal of this work was to analyze prospectively the occurrence of undesirable sequelae in patients undergoing sentinel lymph node biopsy as an isolated intervention in the axillary fossa. This prospective observational study was conducted on a group of 104 women. Patients were examined on five occasions: one day before the procedure, one day after the procedure, one month, three months, and six months after the procedure. At every stage of the study they were assessed for tactile sensation, range of motion in the shoulder joint, upper limb circumference, sensation abnormalities, winged scapula sign, and pain severity according to Visual Analogue Scale (VAS). In the study group we observed statistically significant differences, such as limited mobility in the shoulder joint (*p* ≤ 0.01), gradual increase in limb circumference on the operated side (*p* < 0.01) and pain (*p* ≤ 0.01). Despite relatively low invasiveness of the procedure, sentinel lymph node biopsy is not entirely devoid of the risk of undesirable sequelae.

## Introduction

Breast cancer is the most common type of cancer in females. Undesirable adverse sequelae unrelated to the neoplastic process itself may appear as a consequence of both surgical as well as adjuvant treatment. Such sequelae may be classified as early or late. Early sequelae include: local inflammation, surgical site infection, lymphatic cysts, or pain on the operated side, while late sequelae may present as: neuropathic symptoms in the operated area, fibrosis and contractures of the shoulder joint and shoulder girdle muscles, reduced muscle strength in the limb and shoulder, lymphedema of the breast and upper limb, shoulder joint and upper limb deformations on the operated side, winged scapula, or disrupted posture [1–4].

There are numerous reports comparing the undesirable sequelae among patients after sentinel lymph node biopsy or requiring axillary lymphadenectomy. According to the available literature the SLNB (sentinel lymph node biopsy) procedure is safer, characterized by lower complication rates, as well as lesser severity of those complications [5–19]. The ALMANAC trial has shown that SLNB is associated with less shoulder and arm morbidity, and better quality of life than standard lymph node dissection [10, 11]. Current studies, majority of which are questionnaire-based, show that SLNB is not completely devoid of complications [12–16]. The ALMANAC study has reported that 12-month after surgery, the risk of lymphedema and the area of sensory loss weren't statistically significantly lower for SLNB than for standard lymph node dissection [10].

However, only a few research studies are based on objective parameters.

The goal of this work was to prospectively analyze the undesirable effects of the procedure among patients subjected to sentinel lymph node biopsy as a sole procedure in the axillary fossa. In this work, we also analyzed the possibility of unwanted effects occurring in relation to selected sociodemographic and clinical characteristics.

## Material and Methods

It was a prospective observational study approved by the Bioethical Committee at the Collegium Medicum in Bydgoszcz, Poland (no. KB 213/2012). It was conducted from April 2013 to December 2014 and included 200 consecutive patients with breast cancer (patients without axillary lymph node metastases found during preoperative diagnostics – cN0). All patients were referred for sentinel lymph node biopsy (SLNB). Ninety-six patients did not consent to participate in the study or failed to fulfill the inclusion criteria. Ultimately, the analyzed group comprised 104 patients.

All patients included in the study were hospitalized at the reference center specializing in surgical treatment of malignant breast tumors. The number of surgical procedures involving primary breast cancers performed by each surgeon exceeded 100 per year.

Radioisotope technique was used to identify the sentinel lymph node. Lymphoscintigraphy was performed using ^99m^Tc radionuclide-labeled albumin preparation (activity of 75–100 MBq) (Nanocoll). Isotopic marker was administered intradermally at the breast areolar margin (within the quadrant where primary lesion was located) about 2–3 h before surgery. A compact, portable gamma radiation detector (devices: Crystal Prob, Crystal Photonics, USA and NeoProbe, Autosuture, USA) was used to intraoperatively identify the areas of increased radiolabel uptake in the axillary fossa and to measure radiation levels.

The lymph node exhibiting the highest level of radiation was identified as the sought sentinel lymph node. According to the “10% principle” put forward by Martin et al. [17], lymph nodes exhibiting elevated radiolabel accumulation exceeding 10% of radiation dose identified for the sentinel lymph node (secondary lymph nodes) were also excised.

Inclusion criteria were as follows:

- patients with sentinel lymph nodes and secondary nodes located in the lower part of the axilla (acc. to Berg’s classification),
- patients referred for breast-conserving (breast quadrantectomy) and axillary lymph node- sparing treatment (sentinel lymph node biopsy),
- patients who provided informed consent to participate in the study,
- patient age: 25–70 years.

Exclusion criteria were as follows:

- patients referred for breast amputation,
- change of the originally planned surgical procedure during the diagnostic process (or necessity of broadening the scope of surgical treatment),
- history (or occurrence during the diagnostic process) of upper limb/shoulder girdle trauma.

In order to assess for the presence and severity of undesirable treatment sequelae patients qualified for the analysis were subject to examination according to the following protocol:

- initial evaluation 0: one day before surgery – assessment of baseline status,
- follow-up evaluation I: one day after surgery,
- follow-up evaluation II: one month after surgery,
- follow- up evaluation III: 3 months after surgery,
- final evaluation IV: 6 months after surgery.

Patients who were included in the study were subject to the same scheme of adjuvant treatment (breast radiotherapy as a part of breast-conserving treatment and adjuvant chemotherapy).

Evaluations were conducted according to the following scheme:

- filling out a questionnaire of our own design (age, employment, address),
- gathering of clinical data (cancer TNM (Tumor, Nodes, Metastasis) staging, tumor location, number of nodes removed during SLNB, adjuvant chemotherapy scheme),
- measurements of body height and weight with BMI (Body Mass Index) calculation,
- assessment of upper limb range of motion (flexion, extension, external and internal rotation, abduction) – measurements were performed using a goniometer according to standard norms, results were recorded in degrees
- measurements of upper limb circumference were performed using measuring tape on three levels: I – 10 cm above the lateral epicondyle of humerus, II – 10 cm below the lateral epicondyle of humerus, III – in the mid-metacarpal region (without a thumb),
- examinations of tactile sensation were performed with the monofilament test: the test was performed in the upper limb (arm and forearm regions), in the axillary fossa, thorax, and on the scapula; presence of sensation abnormalities was denoted as “1” and lack thereof as “0,”
- evaluation for the winged scapula sign – during upper limb flexion (diverging of the medial edge of scapula from the chest corresponded to dysfunction of the anterior serratus muscle, which is innervated by the long thoracic nerve),
- pain evaluation using the VAS (Visual Analogue Scale) scale: subjective assessment of the intensity of pain measured on a ten-point scale: the value of “0” denoted lack of pain, while the value of “10” was interpreted as the greatest pain the patient could imagine

Both the affected and the non-affected sides were evaluated.

## Statistical Analysis

The STATISTICA 10PL software was used for statistical analysis. Statistical evaluation of the collected data began by checking for normality of distribution of variables. The Shapiro-Wilk test was used for that purpose. We used the repeated measures ANOVA test in order to compare selected groups with regard to the variables characterized by normal distributions. The chi^2^ independence test was performed to investigate the relationship between belonging to a group and the impact of belonging to a group on categorical variables. In all cases, the level of statistical significance was established at *p* ≤ 0.05.

## Results

Study group was characterized with respect to sociodemographic and clinical characteristics; the results are presented in Table 1.

**Table 1:** Analysis of clinical and sociodemographic features in the studied group of patients

*N* = 104	Descriptive parameters
Age	M = 55.4; SD = 9.31
Weight	M = 69.6; SD = 13.1
Height	M = 1.62; SD = 0,05
BMI	M = 26.5; SD = 5.0
Tumor size (clinical assessment – mm)	M = 14.6: SD = 6.64
Clinical staging of the disease	
- T1N0M0	81 (78%)
- T2N0M0	23 (22%)
Tumor location	
- outer quadrant	78 (75%)
- inner quadrant	19 (18.3%)
- central location	7 (6.7%)
Number of removed lymph nodes	
- 1	25
- 2	40
- 3 and more	39
Education level	
- vocational	39 (37.5%)
- secondary education	46 (44.2%)
- higher	19 (18.3%)
Employment status	
- unemployed	4 (3.9%)
- retired/social security	50 (47.1%)
- actively working	51 (49%)

BMI- Body Mass Index; N - number of patients; M - arithmetic mean; SD - standard deviation

In the studied group of patients we analyzed the range of motion in the shoulder joint, upper limb circumference, presence of winged scapula sign, and sensation in the healthy upper limb. Results are demonstrated in Table 2.

**Table 2:** Comparison of the range of motion in the shoulder joint, limb circumferences at various levels, and other undesirable effects of surgery observed in the upper limbs on the affected and the healthy side

Time point		1 day before surgery	1 day after surgery	1 month after surgery	3 months after surgery	6 months after surgery	Value of calculated probability
Examined parameters							
Movement in the shoulder joint (degrees)							
Flexion	KO	90.0	83.3	86.9	87.1	87.3	NS
	KZ	90.0	89.7	90.0	90.0	89.5	NS
Extension	KO	37.7	33.1	34.4	35.1	35.5	*p* ≤ 0.01
	KZ	37.7	37.6	37.7	37.8	37.8	NS
External rotation	KO	77.8	63.7	70.0	71.5	72.9	*p* ≤ 0.01
	KZ	78.8	78.5	78.8	79.0	79.1	NS
Internal rotation	KO	74.1	68.0	67.4	68.9	69.3	*p* ≤ 0.01
	KZ	72.8	72.9	72.7	73.0	72.9	NS
Abduction	KO	89.7	82.4	86.7	87.4	87.8	*p* ≤ 0.01
	KZ	89.9	89.9	89.9	89.9	89.0	NS
Measurements of upper limb circumference (cm)							
Circumference 1	KO	28.3	28.4	28.7	28.8	28.9	p < 0,001
	KZ	28.2	28.2	28.2	28.4	28.4	NS
Circumference 2	KO	23.6	23.9	23.9	24.0	24.1	*p* = 0.0000
	KZ	23.78	23.74	23.73	23.71	23.65	NS
Circumference 3	KO	18.9	19.0	19.1	19.2	19.2	*p* ≤ 0.014
	KZ	19.0	19.0	18.9	18.9	19.0	NS
Other undesirable sequelae							
Winged scapula sign (number of patients)	KO	0	4	3	3	3	
	KZ	0	0	0	0	0	
Sensory abnormalities (number of patients)	KO	0	36	41	40	38	
	KZ	0	0	0	0	0	
Pain assessment (1–10)		0.4	3.2	2.1	1.5	1.2	*p* ≤ 0.01

KO – upper limb on the operated side; KZ – upper limb contralateral to the operated one; NS – statistically insignificant; p – value of calculated probability; Circumference 1 – 10 cm above the lateral epicondyle of humerus; Circumference 2–10 cm below the lateral epicondyle of humerus; Circumference 3 – mid-metacarpal region without the thumb; *p*-value from paired Student’s t-test

In assessment of the studied parameters involving the limb contralateral to the operated one, we found no statistically significant differences between subsequent measurements performed on the subsequent time points.

During the initial evaluation (one day before surgery) we found no statistically significant differences between groups. For the upper limb on the operated side we found highly significant differences between measurements taken at subsequent time points with respect to the range of extension, internal rotation, external rotation and abduction (*p* ≤ 0.01), upper limb circumferences: 10 cm above the lateral epicondyle (*p* = 0.0000), 10 cm below the lateral epicondyle (*p* = 0.0000), and in the mid-metacarpal region (without the thumb) (*p* ≤ 0.014).

One day after surgery 4 people presented with winged scapula. In 3 persons it persisted at the following stages of assessment. Sensation abnormalities were identified one day after surgery in 36 patients, one month after surgery in 41 patients, in 40 patients disturbances persisted at 3 months after surgery, while after half a hear they were still present in 38 studied subjects.

The undesirable sequelae found to present statistically significant differences were further analyzed by taking into account additional independent factors, such as: age, education, employment status, TNM classification, BMI, number of lymph nodes removed during SLNB and location of the tumor. Results are shown in Table 3.

**Table 3:** Comparison of differences between mean values of range of motion, limb circumference, assessment of pain and sensory abnormalities on the operated side depending on sociodemographic and clinical factors

Characteristics		Age	Education	Employment status	cTNM	BMI	Number of removed lymph nodes	Tumor location
Examined parameters								
Extension		0.2	0.5	0.9	0.5	0.3	0.2	1.0
External rotation		0.08	**0.03**	0.65	0.96	**0.03**	0.06	0.06
Internal rotation		0.41	0.73	**0.05**	0.16	0.52	0.24	0.97
Abduction		0.86	0.68	**0.01**	0.49	0.31	0.81	0.65
Upper limb circumferences at various levels	1	0.3	0.24	0.56	0.23	0.73	0.54	0.61
	2	**<0.014**	0.94	0.12	0.86	0.89	0.36	**0.04**
	3	0.48	0.07	0.99	0.24	**0.05**	**0.02**	0.16
Winged scapula		0.43	0.39	0.86	**0.04**	0.04	0.3	0.25
Sensory assessment		0.08	0.99	0.06	0.77	0.44	**0.02**	0.78
Pain assessment		0.59	0.40	0.32	0.94	0.55	0.08	0.78

cTNM – TNM classification; BMI – Body Mass Index, upper limb circumference: 1–10 cm above the lateral epicondyle of humerus, 2–10 cm below the lateral epicondyle of humerus, 3 – mid-metacarpal region without the thumb; numbers in the table correspond to the *p* value; *p*-value from paired Student’s t-test

No statistically significant influence of independent factors was noted on the range of extension in the shoulder joint, pain assessment using VAS scale and upper limb circumference measured 10 cm above the lateral epicondyle of humerus.

We identified a statistically significant influence of the education level and body mass index (BMI) on a temporal trend in the range of the external rotation in the shoulder joint. Individuals with higher education were characterized by greater range of external rotation in the shoulder and the greatest difference was apparent 6 months after surgery (higher vs. vocational education: 77.6 ± 2.1 vs. 71.7 ± 1.8, respectively). Women with higher BMI values presented with lower range of motion at every stage of the study.

A statistically significant relationship was found between employment status and a temporal trend in the range of internal rotation and shoulder abduction. Actively working patients had greater ranges of internal shoulder rotation. Age and tumor location significantly influenced the temporal trend of upper limb circumference measured 10 cm below the lateral epicondyle.

A similar influence on the temporal trend of measurements of mid-metacarpal circumference was noted in case of BMI and number of lymph nodes removed during SLNB. Obese individuals were characterized by greater growth dynamics of mid-metacarpal circumference at all stages of the study. We noted larger mid-metacarpal circumferences in patients with 3 or more removed lymph nodes. Observed differences were the greatest 6 months after surgery (one lymph node removed: 19.0 ± 0.2 cm; two lymph nodes removed: 19.1 ± 0.2 cm; three or more lymph nodes removed: 19.3 ± 0.2). Winged scapula was identified in women with BMIs exceeding 30 kg/m^2^ and in cases of primary tumors more than 2 cm in diameter (cT2).

Number of removed lymph nodes significantly affected the temporal trend of sensory abnormalities in studied subjects. Disrupted sensation occurred at every stage of the study in patients with three or more lymph nodes removed during SLNB.

## Discussion

Our study demonstrated that the sentinel lymph node biopsy is not completely free of possible undesirable sequelae. We noted statistically significant differences with regard to compared ranges of active limb motion on the operated side (rotation, extension, and abduction), limb circumference measurements on the operated side, sensory abnormalities, presence of winged scapula, or pain. We also noted a relationship between the number of lymph nodes removed during SLNB or BMI and the occurrence and severity of undesirable effects of treatment, such as increased upper limb circumference or sensation abnormalities.

The majority of analyses evaluating the occurrence of undesirable treatment sequelae in patients subjected to isolated surgical intervention in the axillary fossa were based on questionnaires [12–16], allowing only subjective assessment of symptoms. In contrast to those studies, one of the aims of our study was to find a way to collect the data for analysis in a fully objective manner.

In our study we found significantly reduced range of shoulder motion on the operated side (demonstrated differences were statistically significant). Similar results concerning patients treated for breast cancer were obtained in the analyses from other centers. Limitation of the range of shoulder motion was noted particularly with respect to flexion and abduction [19]. It may be caused by intraoperative irritation or damage to the axillary nerve.

Our studies revealed a correlation between patient education and the degree of loss of external rotation range on the operated side. Similar observations were reported by Hack et al. [16].

As demonstrated Purushotham et al., in the majority of cases impaired shoulder mobility improved within short time from surgery [13]. Our studies demonstrated failure to completely restore the range of limb motion in the shoulder joint to the values from before the operation, although we found a clear tendency toward diminishing of motor deficits over time the analyzed group of patients.

Lymphedema is another potential undesirable consequence of sentinel lymph node removal. Patients subjected to axillary fossa lymphadenectomy or sentinel lymph node biopsy are at risk of edema even many years after surgery [20–25]. During the follow-up period of our study we noted gradual increase in upper limb circumference on the operated side on all examined levels. However, they were not characteristic of lymphatic edema.

According to Peitinger et al. sentinel lymph node biopsy may be associated with low-grade pain [19]. In the analyzed clinical material the most severe pain was observed on the first day after the procedure. Its intensity decreased with time and after 6 months it amounted to a mean of 1.2 points on a 10-point VAS scale (differences were statistically significant).

Patients treated for breast cancer may also experience sensory abnormalities and associated symptoms [26–28]. In our analysis, we found sensory abnormalities in nearly 40% of subjects. In the majority of cases they persisted throughout the study.

In our studies we found a correlation between BMI and upper limb circumference on the operated side (mid-metacarpal measurements), which is concordant with the results of other authors [29]. The rationale behind this finding is still unclear. On one hand, it may be related to greater weight of the limb and large amount of subcutaneous tissue. On the other hand, it indicates that more invasive intervention in the surgical field is necessary in case of obese patients (due to greater amount of subcutaneous and adipose tissue) in order to find and remove the sentinel lymph node [30]. However, other studies question the relationship between BMI value and the risk of limb edema [31, 32].

As demonstrated in studies by Kootstra et al., the number of removed lymph nodes is another important factor that might influence the occurrence of complications among breast cancer patients undergoing SLNB. Our results confirm the presence of such a correlation [32]. The greatest differences with regard to the loss of limb mobility compared to other study groups were observed in patients with three or more lymph nodes removed.

Available literature lacks reports analyzing the influence of the number of removed lymph nodes on the occurrence of sensory abnormalities and the severity of pain. As demonstrated in our study, the number of removed lymph nodes may affect sensory disturbances among patients after SLNB. In our study group in the majority of cases such abnormalities occurred when 3 or more lymph nodes were removed.

## Conclusion

Despite limited invasiveness of the procedure, sentinel lymph node biopsy is not completely devoid of the risk of undesirable sequelae. Observed side effects of therapy may involve deficits of shoulder joint mobility, sensory abnormalities, or complications related to nerve damage (sensory disturbances, winged scapula). Their severity may be related to the extent of surgical intervention (number of lymph nodes removed during SLNB), or may depend on other factors (BMI value).

Identified adverse effects of surgical treatment warrant considering physiotherapy in patients undergoing sentinel lymph node biopsy.
